# Continuous, Strong, Porous Silk Firoin-Based Aerogel Fibers toward Textile Thermal Insulation

**DOI:** 10.3390/polym11111899

**Published:** 2019-11-18

**Authors:** Haiwei Yang, Zongqian Wang, Zhi Liu, Huan Cheng, Changlong Li

**Affiliations:** 1 School of Textile and Garment, Anhui Polytechnic University, Wuhu 241000, China; yhwcadillac@163.com (H.Y.); chenghuanbg@126.com (H.C.); fangzqh@126.com (C.L.); 2 CECT Wuhu Diamond Aircraft Manufacture Co., LTD., Wuhu 241000, China

**Keywords:** aerogel fiber, silk fibroin, thermal insulation properties, multiscale pores, hollow fiber

## Abstract

Aerogel fiber, with the characteristics of ultra-low density, ultra-high porosity, and high specific surface area, is the most potential candidate for manufacturing wearable thermal insulation material. However, aerogel fibers generally show weak mechanical properties and complex preparation processes. Herein, through firstly preparing a cellulose acetate/polyacrylic acid (CA/PAA) hollow fiber using coaxial wet-spinning followed by injecting the silk fibroin (SF) solution into the hollow fiber, the CA/PAA-wrapped SF aerogel fibers toward textile thermal insulation were successfully constructed after freeze-drying. The sheath (CA/PAA hollow fiber) possesses a multiscale porous structure, including micropores (11.37 ± 4.01 μm), sub-micron pores (217.47 ± 46.16 nm), as well as nanopores on the inner (44.00 ± 21.65 nm) and outer (36.43 ± 17.55 nm) surfaces, which is crucial to the formation of a SF aerogel core. Furthermore, the porous CA/PAA-wrapped SF aerogel fibers have many advantages, such as low density (0.21 g/cm^3^), high porosity (86%), high strength at break (2.6 ± 0.4 MPa), as well as potential continuous and large-scale production. The delicate structure of multiscale porous sheath and ultra-low-density SF aerogel core synergistically inhibit air circulation and limit convective heat transfer. Meanwhile, the high porosity of aerogel fibers weakens heat transfer and the SF aerogel cellular walls prevent infrared radiation. The results show that the mat composed of these aerogel fibers exhibits excellent thermal insulating properties with a wide working temperature from −20 to 100 °C. Therefore, this SF-based aerogel fiber can be considered as a practical option for high performance thermal insulation.

## 1. Introduction

Aerogel materials, with ultra-low density, large surface area, and continuous three-dimensional nanoporous network structure properties [1], have been widely used in various applications, such as environmental treatment [2,3,4,5], catalytic carrier [6,7], energy storage device [8,9], flexible electronic device [10], and thermal insulation [11,12]. Currently, aerogels can be prepared from a variety of materials, such as silica, metal/metal oxides, carbon-based materials, and organic materials [13].

Among them, silk fibroin (SF) aerogels made by silkworm cocoons have advantages in many industrial applications due to its low cost, widespread resources, excellent biocompatibility, and biodegradability. On the one hand, SF is one of the least thermally conductive materials. The SF-based aerogel has a low thermal conductivity of only 0.026 W/m·K [14], equivalent to air at ambient conditions (0.026 W/m·K) [15,16], indicating the excellent thermal insulation properties. On the other hand, aerogels used to thermal insulation have the following merits: (a) the ultra-high porosity significantly reducing thermal conduction; (b) aerogel cellular walls effectively suppressing thermal radiation; and most importantly, (c) the aerogel’s pore sizes are close to or less than the mean free path of the gas molecule in the air (ca. 70 nm), effectively weakening thermal convection [16,17]. Therefore, the SF-based aerogels combine the SF low thermal conductivity and aerogel’s superior properties, indicating promising potential in thermal insulation areas.

However, reported SF aerogels always show poor mechanical properties [14,18]. As a result, few literatures are found to study the SF-based aerogel fiber. Frontier researches have shown that aerogels can be easily processed and used in combination with other flexible, stretchable materials such as textiles for thermal insulation [19,20]. Nevertheless, the aerogel layers always lose their insulating capacity when their structures are damaged by compression [20]. Therefore, it is recommended that shaping 3D aerogels into one-dimensional fibers may endow some desired properties, such as favorable flexibility, unique structure, and further design possibility.

To date, many efforts have been devoted to develop aerogel fibers. For example, Zhu et al. [21] successfully prepared high surface area hollow silica aerogel fibers by wet spinning using water glass and dilute sulfuric acid as spinning solution and coagulation bath, respectively. Karadagli et al. [22] also used wet spinning to prepare the porous cellulose aerogel fibers with a diameter of about 500 µm and porosity from 93% to 99%. The resulting fibers exhibit better heat resistance than cotton cloth under space flight conditions. In addition, inspired by the polar bear hair, Cui et al. [23] used the freeze spinning method to achieve continuous, large-scale preparation of porous silk fibroin fibers. The resulting fibers show excellent thermal insulation properties, suggesting potential application in smart textiles for effective personal thermal management. Unfortunately, these aerogel fibers above suffer from generally poor mechanical properties or complex preparation processes. To enhance aerogel fibers performance, aqueous liquid crystal graphene oxide gel was wet-spun into liquid nitrogen, and then lyophilized to prepare strong aerogel fibers [24]. However, coagulation in liquid nitrogen and the rigidity of graphene oxide aerogel fibers make continuous fiber production more challenging. Therefore, high performance with acceptable mechanical property and continuous fabrication process are greatly desired in aerogel fiber construction and practical application.

In the present study, the multiscale porous cellulose acetate/polyacrylic acid (CA/PAA) hollow fibers were firstly prepared by the wet coaxial spinning method. Then, the SF solution (1.4 wt. %) was injected into the hollow fiber, followed by freezing/freeze-drying to prepare continuous, high-strength and porous CA/PAA-wrapped SF aerogel fibers. We aim to investigate (1) the construction process of the SF-based aerogel fibers; (2) the microstructure, mechanical properties of SF-based aerogel fibers; (3) the thermal insulation properties of the SF-based aerogel fibers; and (4) the proposed thermal transfer mechanism of the SF-based aerogel fibers. The results show that the multiscale porous CA/PAA hollow fiber can be used as a template and protective layer for the SF aerogel core. The unique structure of porous CA/PAA hollow fiber and the ultra-low-density SF aerogel core synergistically weakens the three heat transfer modes (including thermal convection, thermal conduction, and thermal radiation), resulting in gratifying thermal insulating properties with wide working temperatures ranging from −20 to 100 °C.

## 2. Experimental

### 2.1. Materials and Chemicals

The raw silk was supplied by Qingyang Sanfang Silk Co., Ltd. (Chizhou, China). Anhydrous calcium chloride (CaCl_2_), anhydrous ethanol (C_2_H_5_OH), urea, poly (acrylic acid) (PAA, *M*_n_ = 4000 kDa), and dimethylacetamide (DMAc) were all purchased from Aladdin Reagent Co., Ltd. (Shanghai, China). Cellulose acetate (CA, *M*_n_ = 50 kDa) was obtained from Sigma Aldrich Co., Ltd. (Shanghai, China). All reagents used in this research were of analytical grade and without further treatment.

### 2.2. Preparation of SF Solution

SF solution was extracted from raw silk through a slightly modified standard procedure reported by Wang et al. [25]. Briefly, the raw silk was firstly degummed with a solution containing 8 mol/L of urea powder with a liquor-to-silk ratio of 30:1 for 3 h at 90 °C. After degumming treatments, the resulting raw products were rinsed thoroughly with distilled water and dried in an oven at 40 °C. Then, the degummed silk was dissolved in a ternary solvent of CaCl_2_/C_2_H_5_OH/H_2_O with the molar ratio of 1:2:8 for 2 h at 80 °C. The SF solution was dialyzed continuously using dialysis bag (Union Carbide Corporation, cutoff 8–14 kDa) with distilled water for 72 h. Moreover, the water was changed every 4 h to remove CaCl_2_ and C_2_H_5_OH. The conductivity of dialysis water was tested and the value was less than 0.8 μS/cm. The concentration of SF solution after dialysis was diluted to 1.4 wt. % and stored at 4 °C for SF-based aerogel fiber preparation.

### 2.3. Preparation of CA/PAA Hollow Fibers

PAA (0.2 g) was first vigorously stirred in DMAc (13.1 g) for 1 h. Then, the CA (2.3 g) was added to prepare 16 wt. % concentration of CA/PAA (23:2 w/w) mixture and stirred for 4 h. Each of the core (water) and shell solutions (CA/PAA in DMAc) was loaded into 10 mL syringes and spun through respective outer (17 gauge (G)) and inner (23 G) stainless steel needles at the same 300 µL·min^−1^ rate using ZS100 syringe pumps (Baoding Chuangrui Precision Pump Co., Ltd., Baoding, China). The fibers were continuously spun into a water bath at room temperature and collected on a 50 mm diameter winding spool at 1.8 to 2 m·min^−1^ line speed. The as-spun fibers were soaked in a water bath for 15 h to remove residual DMAc, frozen at −20 °C for 15 h, lyophilized at −50 °C for 2 days using the LGJ-10 Vacuum freeze dryer (Beijing Song Yuan Hua Xing Technology Develop Co., Ltd., Beijing, China). Thus, the CA/PAA hollow fibers were prepared.

### 2.4. Preparation of CA/PAA-Wrapped SF Aerogel Fibers

The as-prepared SF aqueous dispersion was loaded into a 10 mL syringe firstly, then injected into the CA/PAA hollow fibers with a length of 4 m using a ZS1001 syringe pump at 300 µL·min^−1^. The CA/PAA hollow fibers containing SF aqueous dispersion were then frozen (−20 °C, 15 h) and lyophilized (−50 °C, 2 d) to yield the polymer-wrapped SF aerogel fibers.

### 2.5. Measurement and Characterization

*Viscosity measurement:* The RST type rheometer (Brookfield, New York, NY, USA) equipped with a RCT-75-1 cone spindle with a diameter of 75 mm and a gap of 0.046 mm was used for the rheological property test. The shear rate of the rheometer was set to linearly increase from 0 to 800 s^−1^ [26]. The test was conducted at a constant temperature of 20 °C.

*Characterization on micromorphology:* The cross section, inner and outer surface micromorphology of fibers were measured using S-4800 scanning electronic microscopy (Hitachi, Tokyo, Japan) at an acceleration voltage of 5 kV after sputtering with gold. The diameter of the microvoids, sub-micron pores, and nanopores on the inner and outer surface of the CA/PAA sheath were measured and averaged from 30 pores by the Image J program. At the same time, based on the diameter data of these 30 pores, their diameter distribution figures were plotted using the GraphPad Prism 8 software.

*Density and porosity measurements*: The cross-section dimension of the fibers was measured from SEM images, and their mass was measured by a balance with 0.1 mg resolution, to calculate the fiber density (*ρ_f_)*. The porosity of the fibers (*P_f_*) was calculated as *P_f_* = 1−*ρ_f_*/*ρ_b_*, where *ρ_b_* is the bulk density of silk fibroin and equals 1.464 g/cm^3^ [27].

*Mechanical properties measurements:* The mechanical properties of hollow CA/PAA fiber, CA/PAA-wrapped SF aerogel fibers were measured by a 5543 Instron universal testing machine (Instron, Norwood, MA, USA). The gage length was 20 mm with a strain rate of 1 mm/min. The test samples were 5 cm in length. The tensile strength, Young’s modulus, and elongation were collected and averaged from at least 5 samples for each formulation.

*Characterization on thermal insulation properties*: The representative test sample was constructed containing layers of tightly packed fibers (each layer contains 20 unidirectional fibers with the length of 5 cm). Then the samples were placed on a hotplate, and the sample surface temperature, when the heating plate was heated from 25 to 100 °C, was measured using a thermal couple connected to a temperature controller (Shenzhen Yisheng Victory Technology Co., Ltd., Shenzhen, China). In order to simulate a cold condition, the samples were also placed on the metal substrate with a 2.5 cm thick slab of dry ice underneath. The samples’ surface temperature was monitored and recorded by a thermocouple. The corresponding absolute temperature difference (|Δ*T*|) between the sample surface and the heat or cold source were calculated to characterize the thermal insulation properties. During measurements, the ambient temperature was around 23 °C. The temperature values were recorded when the fiber surface temperature was stable. The thermal conductivity of the aerogel fibers was measured using a TC3010L thermal conductivity meter (Xi’an Xiatech Electronic Technology Co., Ltd., Xi’an, China) [28].

## 3. Results and Discussions

### 3.1. Construction of CA/PAA Hollow Fibers and CA/PAA-Wrapped SF Aerogel Fibers

**Figure 1:**
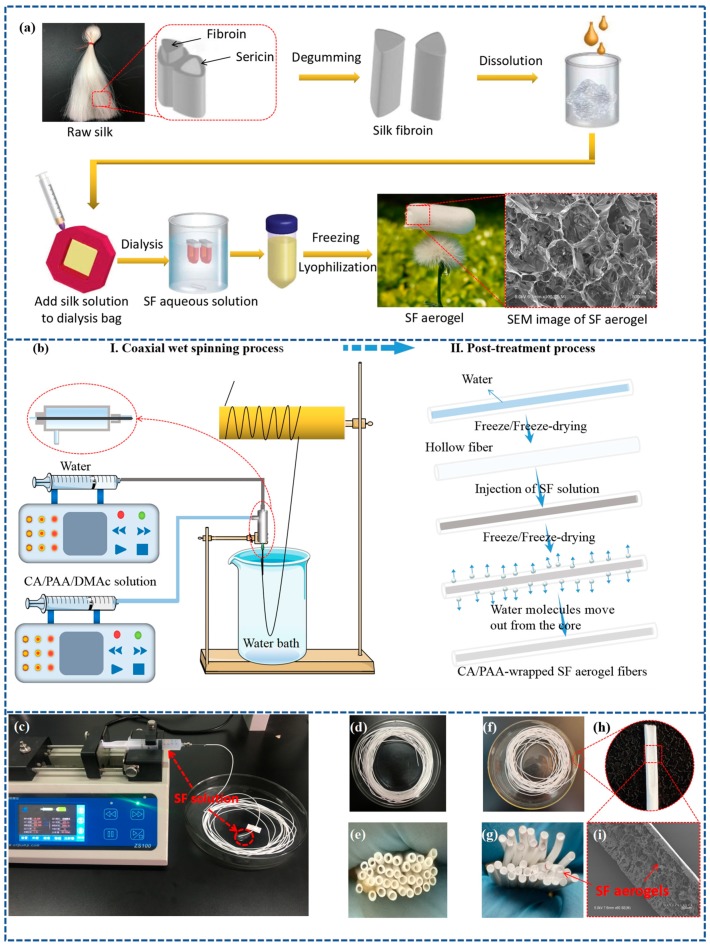
(**a**) Typical preparation process of 3D bulk silk fibroin (SF) aerogel. (**b**) Schematic diagram of coaxial wet-spinning of cellulose acetate/polyacrylic acid (CA/PAA) hollow fiber and post-processes to produce CA/PAA-wrapped SF aerogel fibers. (**c**) Optical image of silk fibroin solution injected into CA/PAA hollow fiber. (**d**,**e**) Optical image of CA/PAA hollow fibers. (**f**–**i**) The CA/PAA-wrapped SF aerogel fibers with arrows pointing at the exposed SF aerogel core of aerogel fiber cross-sections and longitudinal sections.

Figure 1a shows the typical preparation process of SF aerogels. A 3D bulk SF aerogel with low density (13.4 mg/cm^3^) and high porosity (99.08%) was prepared by degumming, dissolving, dialysis, and freeze-drying. As can be seen from Figure 1a, the SF aerogel is highly porous with the pores encased in cellular walls, which significantly reduces the heat transfer [14,29]. Additionally, the SF aerogel cellular wall structure further limits air circulation to reduce thermal convection. Thus, such SF aerogels indicate potential application in thermal insulation. However, 3D bulk SF aerogels with poor mechanical properties are difficult to apply to the thermal insulation of textiles. Because the aerogel structure may be damaged by compression due to its poor mechanical properties, previous literatures integrated aerogels onto textiles to improve their thermal insulation properties [20].

Therefore, an approach was designed to prepare continuous, strong, porous polymer-wrapped SF aerogel fibers for textiles thermal insulation. The polymer sheath can act as processing template and protective layer for external force or environmental damage. Figure 1b shows the coaxial wet-spinning and post-treatment process to produce CA/PAA-wrapped SF aerogel fibers. The spinning nozzle consists of coaxial inner and outer channels constituted by 23 and 17 G needles, respectively. The hollow fiber was first wet-spun through the injection of CA/PAA/DMAc spinning solution in the outer channel and pure water in the inner channel. It should be pointed out that high molecular weight PAA of 4000 kDa was added to CA to increase the viscosity of the spinning solution and enhance its spinnability (Figure S1). The CA/PAA/DMAc solution was then wet-spun into a water coagulation bath in which the water inside and outside could extract DMAc (Movie S1) and induce phase inversion in the sheath, resulting in continuous collection of CA/PAA hollow fiber with a length of more than 8.5 m (Figure 1d). The continuous spinning of uniform fibers demonstrates its potential to scaled-up production.

Subsequently, the as-spun hollow fiber was immersed in deionized water to remove residual DMAc and keep the core filled with water. Thereafter, the hollow fiber with water in the core was frozen (−20 °C, 15 h) and then freeze-dried in a freeze dryer at −50 °C for 2 d to prepare a uniform hollow fiber. The SF aqueous solution (1.4 wt. %) was then injected into the hollow fiber at 300 µL·min^−1^ (Figure 1c and Figure S2a), followed by freezing (−20 °C, 15 h), and then freeze-drying (−50 °C, for 2 d) to form continuous SF aerogel in the core. Figure 1f shows the final CA/PAA-wrapped SF aerogel fibers. As can be seen from cross-section morphology (Figure 1g,h), the hollow fiber core was occupied by the SF aerogel. In addition, from the Figure S2b, the manufacturing process was streamlined to allow continuous production of CA/PAA-wrapped SF aerogel fibers, indicating a potentially practical application.

### 3.2. Morphology of CA/PAA Hollow Fiber and CA/PAA-Wrapped Aerogel Fiber

**Figure 2:**
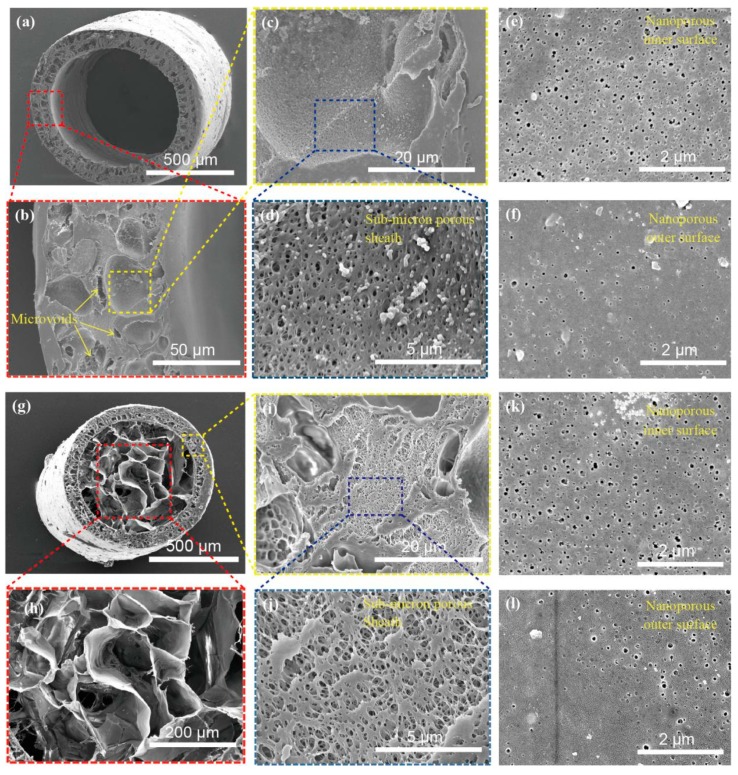
SEM images of hollow and aerogel fibers. (**a**–**d**) The cross-section morphology of CA/PAA hollow fiber with ×80, ×800, ×2.5k, ×10k, respectively; (**e**,**f**) the inner and outer surfaces of the hollow fiber showing nanoporous structures; (**g**) the aerogel fiber cross-section showing porous sheath and core; (**h**) the SF aerogel core of the aerogel fiber; (**i**,**j**) the sheath in aerogel fiber showing similar micro-voids structures as in the hollow fiber sheath; (**k**,**l**) the inner and outer surfaces of the aerogel fiber sheath showing nanoporous structure.

Figure 2a–f presents the SEM images of CA/PAA hollow fibers. As can be seen in Figure 2a, the average outer diameter of CA/PAA hollow fiber is 964.21 ± 13.61 μm with an average wall thickness of 137.02 ± 9.55 µm. The wall shows high porous structures, which are considered to be caused by the combination of phase separation and mass transfer among CA/PAA (polymer), DMAc (solvent) and water (coagulation bath) during phase inversion [30].

**Figure 3:**
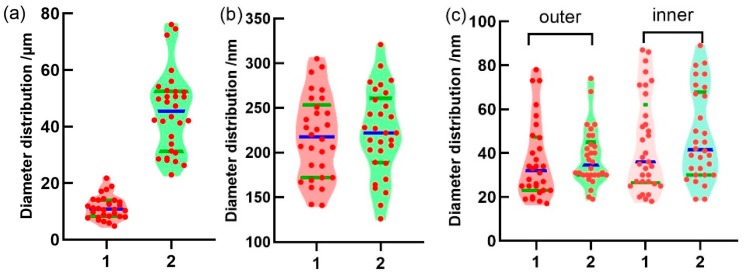
(**a**) Micropore diameter distribution of the hollow fiber sheath and the SF aerogel core. (**b**) Diameter distribution of sub-micron pores in the hollow fiber and aerogel fiber sheaths. (**c**) Diameter distribution of nanopores on the inner and outer walls of hollow fibers and aerogel fibers. 1—CA/PAA hollow fiber, 2—CA/PAA-wrapped SF aerogel fiber.

As shown in Figure 2b and Figure 3a, the diameter of micro-voids in the CA/PAA hollow fiber wall is ranging from several to tens of micrometers (average diameter 11.37 ± 4.01 μm). Further magnifying the SEM image of the hollow fiber, it is observed that the hollow fiber wall also has sub-micron pores with an average diameter of 217.47 ± 46.16 nm (Figure 2c,d) and even smaller nanopores on both inner (44.00 ± 21.65 nm) and outer (36.43 ± 17.55 nm) surfaces (Figure 2e,f). Therefore, the pores in the hollow fiber have three orders, namely, tens of μm micro-voids, hundreds of nm sub-micron pores across the wall, as well as tens of nm nanopores on both the inner and outer surfaces (the pore size distributions are shown in Figure 3a–c). It is apparent that the multiscale porous structure of the hollow fiber is crucial to the water mass transfer and ice sublimation during freeze-drying, promoting the formation of SF aerogels in the core.

The formation of graded pores in the hollow fiber wall may be owing to DMAc–water liquid–liquid demixing and mass transfer during the whole phase separation process [30,31]. The nanopores on the inner and outer surfaces are believed to be formed by rapid solidification or instantaneous stratification of the water inside and outside the fibers [32]. The dense skins limit the solvent outflow to promote droplet nucleation and sub-micron pore formation in the sheath in which the CA/PAA/DMAc mixture phase separates into a solvent-rich or polymer-poor phase [30]. The micropores in the sheath may be formed by two generally accepted mechanisms: (a) diffusion assisted by the Marangoni effect, i.e., mass transfer along the interface between the two fluids due to surface tension gradients; (b) local surface instability, skin rupture, and solvent intrusion [32,33]. The sub-micron pores are likely coming from droplets of the solvent-rich phase, after the solution becoming stable and then growing as a continuous entity.

Subsequently, the SF solution was injected into the CA/PAA hollow fiber, followed by freezing and freeze-drying to prepare CA/PAA-wrapped SF aerogel fibers. The outer diameter of the aerogel fibers is 853.35 ± 19.34 μm and the wall thickness is 110.10 ± 7.10 μm, both of which are smaller than the initial hollow fiber. The shrinkage may be due to the compatibility between the SF and CA dominated sheath during freezing and freeze-drying. The SF aerogels fully occupy the core and show highly porous structure with an average diameter of 19.71 ± 8.53 μm (Figure 2g,h). The average diameter of sub-micron pores in the aerogel fiber sheath is 222.47 ± 47.94 nm (Figure 2i,j), which is similar to the hollow fiber (217.47 ± 46.16 nm). Moreover, the average diameter of the nanopores on the inner and outer surfaces of the aerogel fibers are 47.63 ± 20.45 nm and 37.97 ± 12.86 nm, respectively. (Figure 2k,l). Compared with the hollow fiber, the pore sizes of sub-micron pores and nanopore in the aerogel fiber sheath remain basically unchanged with the core (Figure 3b,c), which further confirms that they have chemical similarity and compatibility.

### 3.3. Textural Properties and Mechanical Properties

The linear density of the hollow and aerogel fiber was measured to be 1.09 and 1.18 mg/cm by the mass of equal length (2.5 m) of CA/PAA hollow fiber and CA/PAA-wrapped SF aerogel fiber (Figure S3), respectively. The overall bulk density and porosity of the porous sheath in the hollow and aerogel fibers was 0.54 and 0.21 g/cm^3^, 58%, and 86%, respectively. Furthermore, the density of the SF core was calculated to be 12.7 mg/cm^3^, which is similar to previous SF aerogel [29,34], further confirming that the SF aerogel core in the hollow fiber was not affected by the sheath.

**Figure 4:**
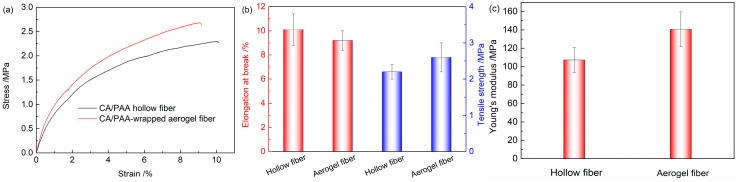
Mechanical properties of CA/PAA hollow fiber and CA/PAA-wrapped SF aerogel fiber: (**a**) stress-strain curve; (**b**) elongation at break and tensile strength; (**c**) Young’s modulus.

The mechanical properties of CA/PAA hollow fibers and CA/PAA-wrapped SF aerogel fibers are presented in Figure 4. Both hollow and aerogel fibers exhibit typical plastic deformation stress-strain curves under tensile loading (Figure 4a) and good flexibility with 10.1 ± 1.3% and 9.2 ± 0.8% elongation at break, respectively. Meanwhile, the tensile strength of the aerogel fiber is 2.6 ± 0.4 MPa, which is higher than that of the hollow fiber (2.2 ± 0.2 MPa). In addition, the aerogel fiber suffers from a higher Young’s modulus of 140.7 ± 18.9 MPa than that for the hollow fiber (107.2 ± 13.5 MPa). Compared to porous silk fibroin fibers [20], the above analysis clearly confirms that the hollow fiber has a protective and strengthening effect in improving the tensile strength and flexibility of the SF aerogel.

### 3.4. Thermal Insulation Properties

The thermal conductivity of CA/PAA-wrapped SF aerogel fibers was measured as 0.031 W/(m·K), which is lower than SiO_2_-based aerogels [35,36,37]. Meanwhile, the thermal conductivity of the aerogel fiber remains competitive among previous aerogel-based thermal insulation materials, including inorganic aerogels (e.g., SiO_2_-based and carbon aerogels) and organic aerogels (e.g., cellulose and SF-based aerogels) (Figure S4). Although some bulk aerogels, such as SiO_2_ aerogels, carbon aerogels, and SF aerogels, exhibit lower thermal conductivity, they suffer from the lower mechanical strength. With both good thermal insulation and mechanical properties which are comparable to the reported Kavlar aerogel fibers [28], the CA/PAA-wrapped SF aerogel fibers show great potential in wearable textile for thermal insulation.

**Figure 5:**
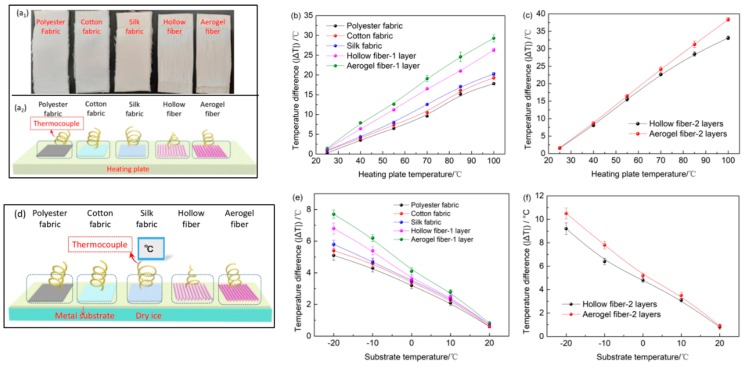
Thermal insulation properties of the hollow and aerogel fiber mats under hot and cold environments. (**a_1_**) Optical photo of samples for testing and (**a_2_**) schematic diagram of the test under hot conditions. (**b**) Temperature difference (|Δ*T*|) of the fiber surface (*T_f_*) and heating plate (*T_h_*) versus the *T_h_* for polyester fabric, cotton fabric, silk fabric, 1-layer hollow fibers and 1-layer aerogel fibers. (**c**) |Δ*T*| of the *T_f_* and *T_h_* versus the *T_h_* for 2-layer hollow fibers and 2-layer coaxial fibers. (**d**) Schematic diagram of the test under cold conditions. (**e**) |∆*T*| of the *T_f_* and cold substrate (*T_s_*) versus the *T_s_* for polyester fabric, cotton fabric, silk fabric, 1-layer hollow fibers, and 1-layer aerogel fibers. (**f**) |∆*T*| of the *T_f_* and *T_s_* versus the *T_s_* for 2-layer hollow fibers and 2-layer coaxial fibers.

The thermal insulation properties of hollow and aerogel fibers under both hot and cold environments were tested and compared with polyester fabrics, cotton fabrics, and silk fabrics with similar thickness. Firstly, 20 fibers were packed tightly and aligned unidirectionally to form a single layer mat with about 1 mm thickness on a heating plate (Figure 5a_1_). The fiber surface temperature (*T_f_*) was measured when the heating plate was heated from 25 to 100 °C using a thermocouple (Figure 5a_2_). The corresponding absolute temperature difference (|∆*T*|) between the fiber surface and the heating plate were plotted against the heating plate temperature (*T_h_*) (higher |∆*T*| indicates better thermal insulation properties). Figure 5b shows that the |∆*T*| of a 1-layer aerogel fiber mat is consistently higher than a 1-layer hollow fiber mat, polyester fabric, cotton fabric, and silk fabric (thickness: 1.10, 1.13, and 1.05 mm, respectively) at any given *T_h_*. As *T_h_* is 100 °C, the aerogel fiber mat temperature increases to 70.7 °C, while the surface temperature of hollow fiber mat, polyester fabric, cotton fabric, and silk fabric reach to 73.7, 82.2, 80.7, and 79.7 °C, respectively, suggesting better thermal insulation properties of aerogel fiber mat than hollow fiber mats and conventional fabrics. For the 2-layers hollow and aerogel fiber mat, owing to the reduced thermal convection, the |Δ*T*| is significantly higher than the 1-layer fiber mat under the same *T_h_* (Figure 5c). At *T_h_* = 100 °C, the |Δ*T*| of the 2-layers aerogel fiber and hollow fiber mat are 38.4 and 33.1 °C, respectively. The thermal insulation property of aerogel fibers is significantly better than hollow fibers, which can be attributed to the SF aerogel core. Recent studies have shown that the thermal insulation property of textiles is determined by many factors. Among them, thermal convection, solid/air thermal conduction, and thermal radiation are the main parameters [22,38,39]. Compared to hollow fibers and conventional fabrics, the thermal convection of aerogel fiber is greatly restricted because air is blocked within micropores, mesopores, and macropores. Meanwhile, the aerogel fiber has high porosity so that its thermal conduction is significantly reduced as air has much smaller thermal conduction than solid. Furthermore, the heat transfer by infrared radiation of the cellular wall in the SF aerogel network was largely reduced [23].

In order to simulate a cold condition, the 1-layer aerogel fiber mat, 1-layer hollow fiber mat, and a similar thickness of polyester fabric, cotton fabric, and silk fabric were placed on top of a metal plate with a 2.5 cm thick slab of dry ice underneath (Figure 5d). At the same time, the fiber surface temperature (*T_f_*) at a metal substrate temperature (*T_s_*) from −20 to 20 °C was measured to calculate the corresponding absolute temperature difference |Δ*T*| between the textile surface and metal substrate (Figure 5e). At *T_s_* = −20 °C, the |∆*T*| of the 1-layer aerogel fiber mat was 7.7 °C, higher than that of the hollow fiber mat and conventional fabrics with a similar thickness. In addition, the |Δ*T*| of the 2-layers aerogel fiber mat was also higher than the 2-layers hollow fiber mat at the same *T_s_* (Figure 5f), further affirming that aerogel fibers have better thermal insulation properties than hollow fibers and conventional fabrics in cold environments. Therefore, aerogel fiber mats have proven to be highly thermally insulating under both hot and cold external circumstance.

**Figure 6:**
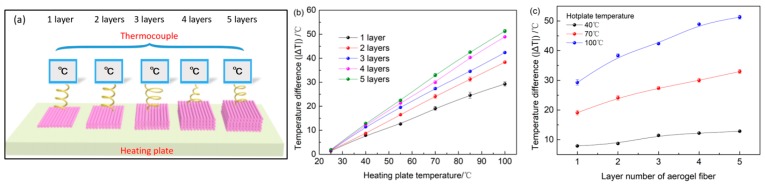
Thermal insulation properties of aerogel fiber mats with different layers. (**a**) Schematic diagram of thermal insulation properties of different layers of aerogel fiber mats. (**b**) The temperature difference (|Δ*T*|) between the fiber surface and heating plate versus the heating plate temperature for different layers of aerogel fiber mats. (**c**) The temperature difference (|Δ*T*|) between the fiber surface and heating plate is plotted against layer number of the aerogel fiber mat.

To investigate the effect of the layer number on the thermal insulation performance, the 1- to 5-layers aerogel fiber mats were placed on top of the heating plate (Figure 6a), respectively. Simultaneously, the *T_f_* of the different layers aerogel fiber mats was measured when the heating plate was heated from 25 to 100 °C. The corresponding |Δ*T*| between the fiber surface and heating plate was calculated, as shown in Figure 6b,c. For the 5-layers aerogel fiber mat, its *T_f_* is as low as 27.1, 37.0, and 48.7 °C, corresponding to |∆*T*| of 12.9, 33.0, and 51.3 °C at *T_h_* = 40, 70, and 100 °C, respectively. Obviously, the greater the number of aerogel fiber mats layer, the lower the rate of heat transfer, indicating that increasing thickness can achieve better thermal insulation performance. Thus, the thermal insulation performance can be adjusted by simply varying the thickness of the aerogel fiber mat. In addition, it can be speculated that the aerogel fiber products can be developed to meet specific needs by varying the diameter of the fibers and adjusting sheath-core proportions.

**Figure 7:**
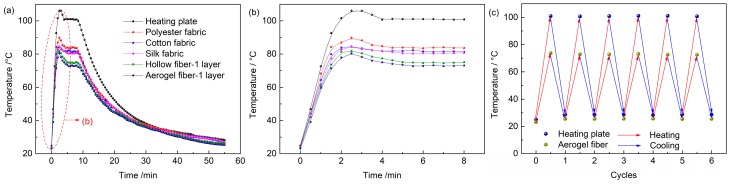
The dynamic thermal insulation properties of different textiles. (**a**) Temperature-time curves of the different textiles. (**b**) Temperature-time curves of the different textiles in the first 8 min. (**c**) When the heating plate is cyclically heated up at a constant rate and air-cooled down between 28 and 100 °C, the aerogel fiber mat surface also cycles but within a much narrower range between 26 and 73 °C.

To further test the dynamic thermal insulation property of the aerogel fiber mat, two thermal couples were attached on both the textile surface and the heating plate. Figure 7a,b shows the dynamic temperature–time curves of heating plates and different textiles. When the heating plate temperature increased from 28 to 100 °C, all textile surfaces heated up but had different response speeds and temperatures, indicating that they possessed different thermal insulation properties. Among them, compared with hollow fibers and other commercial fabrics, the heat transfer rate of aerogel fibers was the smallest. When the temperature of the heating plate reached an equilibrium of 100 °C, the equilibrium temperature on the aerogel fibers mat was the lowest (73 °C). We have also tested the dynamic temperature variation during the heating–cooling cycles for the 1-layer aerogel fiber mat (Figure 7c). While the heating plate cycled between 28 and 100 °C, the aerogel fiber mat surface only cycled with a much narrower range between 26 and 73 °C, further demonstrating the excellent thermal insulation property of aerogel fiber mat.

### 3.5. Heat Transfer Mechanism of CA/PAA-Wrapped SF Aerogel Fiber

**Figure 8:**
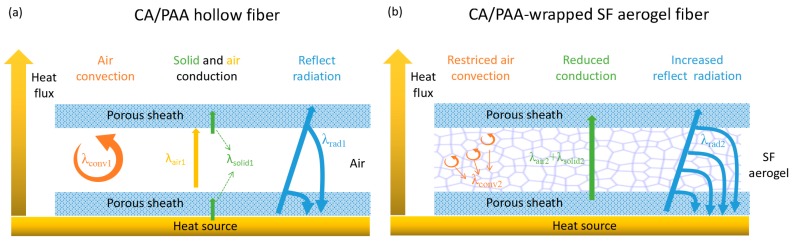
Schematic illustration of the thermal insulation behaviors of the (**a**) hollow and (**b**) aerogel fiber.

Figure 8 illustrates the heat transfer mechanism of both hollow and aerogel fibers. Theoretically, the thermal conductivity (λ_1_) of hollow fibers, λ_1_ can be expressed as [40,41]

λ_1_ = λ_conv1_ + λ_cond1_ + λ_rad1,_ (1)

where λ_conv1_ is the heat transfer by air convection, λ_cond1_ is the heat transfer through the air and solids (CA/PAA) in hollow fibers, and λ_rad1_ is the heat transfer by the radiation. As the SEM analysis of hollow fibers aforementioned, the hollow fiber has a multiscale porous structure with a porosity of 58%, which is extremely important for the overall heat transfer. Thermal convection (λ_conv1_) is significantly reduced thanks to air movement seriously restricted within the multiscale porous structure in the hollow fiber shell. Meanwhile, as air has much smaller thermal conduction than solids (λ_air1_ < λ_solid1_), the thermal conduction (λ_cond1_) of the hollow fibers is dramatically reduced. Furthermore, the reflected radiation (λ_rad1_) of infrared light in the white sheath was significantly reduced because of the large number of solid–air interfaces.

To sum up, these hierarchical porous structures of hollow fibers provide a variety of means to prevent heat transfer. Compared with traditional hollow fiber achieving heat shielding and insulation by trapping air [42], the multiscale pores in the hollow fiber sheath here not only show lighter, more porous, and better thermally insulating property, but also can be used as a processing template for aerogel formation in the core.

The thermal conductivity of the SF aerogel core (λ_2_) can be expressed as [43,44]

λ_2_ =λ_conv2_+ λ_cond2_ + λ_rad2,_ (2)

where λ_cond2_ is the heat transfer via the air and solids (SF) in the aerogel core. λ_conv2_ and λ_rad2_ are the heat transfer by convection and radiant of the aerogel, respectively.

The core of SF aerogel further inhibits the heat transfer mechanism in various ways. Firstly, the cellular network structure and large (tens of μm) pore size in the SF aerogel limit thermal convection by suppressing air circulation. Secondly, SF aerogels have an ultra-high porosity over 99% [14,29], resulting in a significant reduction in the thermal conduction of aerogels as air has much smaller thermal conduction than solid. Finally, compared to the optically transparent polymer films or silica aerogels, the aerogel cellular network structure consisting of SF self-assembled walls prohibits infrared radiation effectively [20,23]. Furthermore, the porous sheath (CA/PAA hollow fiber) wrapping the SF aerogel core also plays several key roles. On one hand, the hollow fibers provide an interior space for filling the aqueous SF aerogel precursor. On the other hand, the multiscale porous structure of the sheath can facilitate sublimation of ice during freeze-drying to form a continuous aerogel core. More importantly, the porous sheath protects the aerogel and its multiscale porous structure helps in restraining air convection. In addition, the porous sheath wrapping SF aerogel can effectively inhibit radiation. Based on the analysis above, the superior thermal insulation of the CA/PAA-wrapped SF aerogel fiber is attributed to the synergistic thermal insulation characteristics of the porous sheath and the aerogel core. In theory, the thermal insulation properties of such polymer-wrapped aerogel fiber may be further improved by designing the microstructure of the core and the sheath; for example, reducing the pore sizes in the sheath to less than the mean free path of air (ca. 70 nm), closing the opened cellular network into enclosed air pockets, or reducing the pore size of the aerogel core while maintaining a high porosity can potentially improve the thermal insulation properties of aerogel fibers. However, the volume ratio of the aerogel core and the sheath may also need to be adjusted to balance density, porosity, and specific for desired thermal insulation properties.

## 4. Conclusions

In summary, continuous, strong, porous CA/PAA-wrapped SF aerogel fibers were successfully fabricated for high-performance thermal insulation textiles by coaxial wet-spinning hollow fiber, injection of SF solution, and a freeze-drying process. The CA/PAA hollow fibers provide the porous sheath, which not only facilitates the formation the SF aerogel core but also protects and endows better mechanical strength to it. The CA/PAA-wrapped SF aerogel fibers exhibit fascinating characteristics of low density (0.21 g/cm^3^), high porosity (86%), and high tensile strength (2.6 ± 0.4 MPa). In addition, due to both the porous sheath and aerogel core, the CA/PAA-wrapped SF aerogel fiber exhibits excellent thermal insulation properties in both cold (−20 °C) and hot (100 °C) conditions. Furthermore, it can be speculated that the thermal insulation property could be adjusted by changing the sheath and core proportion and other parameters. Therefore, such a delicate core–shell structure aerogel fiber provides an alternative approach to developing high-performance wearable thermal insulation materials.

## Supplementary Materials

The following are available online at https://www.mdpi.com/2073-4360/11/11/1899/s1.
