# Supplementary material for: Continuous, Strong, Porous Silk Firoin-Based Aerogel Fibers toward Textile Thermal Insulation
Source: Polymers (Basel). 2019 Nov 18;11(11):1899. doi: 10.3390/polym11111899 (PMC6918396; doi:10.3390/polym11111899)
Supplement: Supplementary file 1 [file polymers-11-01899-s001.zip › SI.pdf]

Continuous, strong, porous silk firoin-based aerogel fibers toward textile thermal insulation

Haiwei Yang[1]， Zongqian Wang[1*] , Zhi Liu[1,2*] , Huan Cheng[1] and Changlong Li[1]

1.School of Textile and Garment, Anhui Polytechnic University, Wuhu, 241000, China;

2.CECT Wuhu Diamond Aircraft Manufacture Co., LTD., Wuhu, 241000, China.

*Corresponding author:

E-mail: wzqkeyan@126.com (Z.Q. Wang), liuzhi@ahpu.edu.cn (Z. Liu)

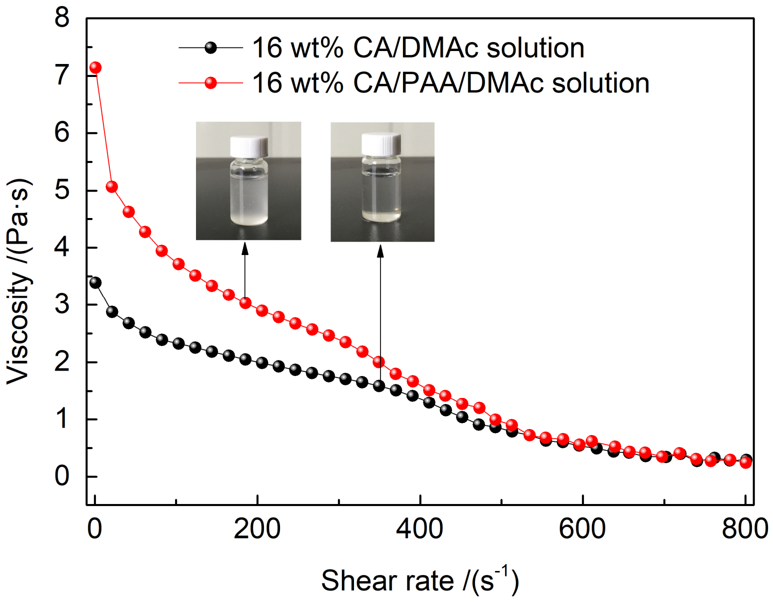

Figure. S1 Rheological curves of 16 wt. % CA/DMAc solution and 16 wt. % CA/PAA/DMAc solution.

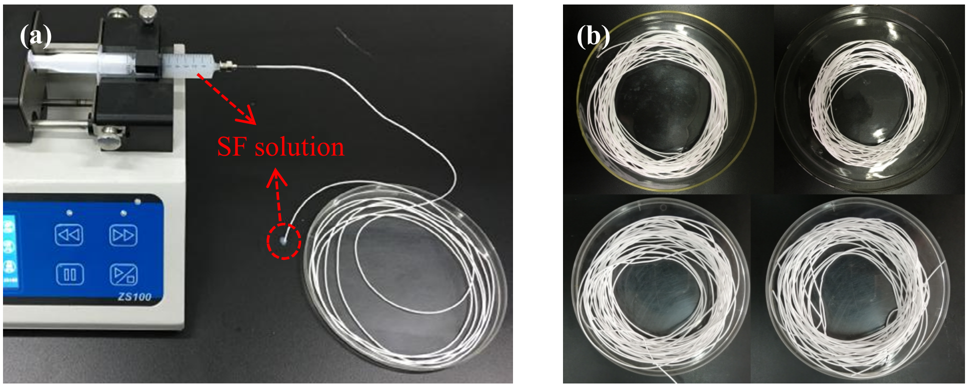

Figure. S2 (a) Filling aqueous SF solution (1.4 wt. %) into a 3 m-length CA/PAA hollow fiber. (b) CA/PAA hollow fibers containing SF solution following freezing and freeze drying.

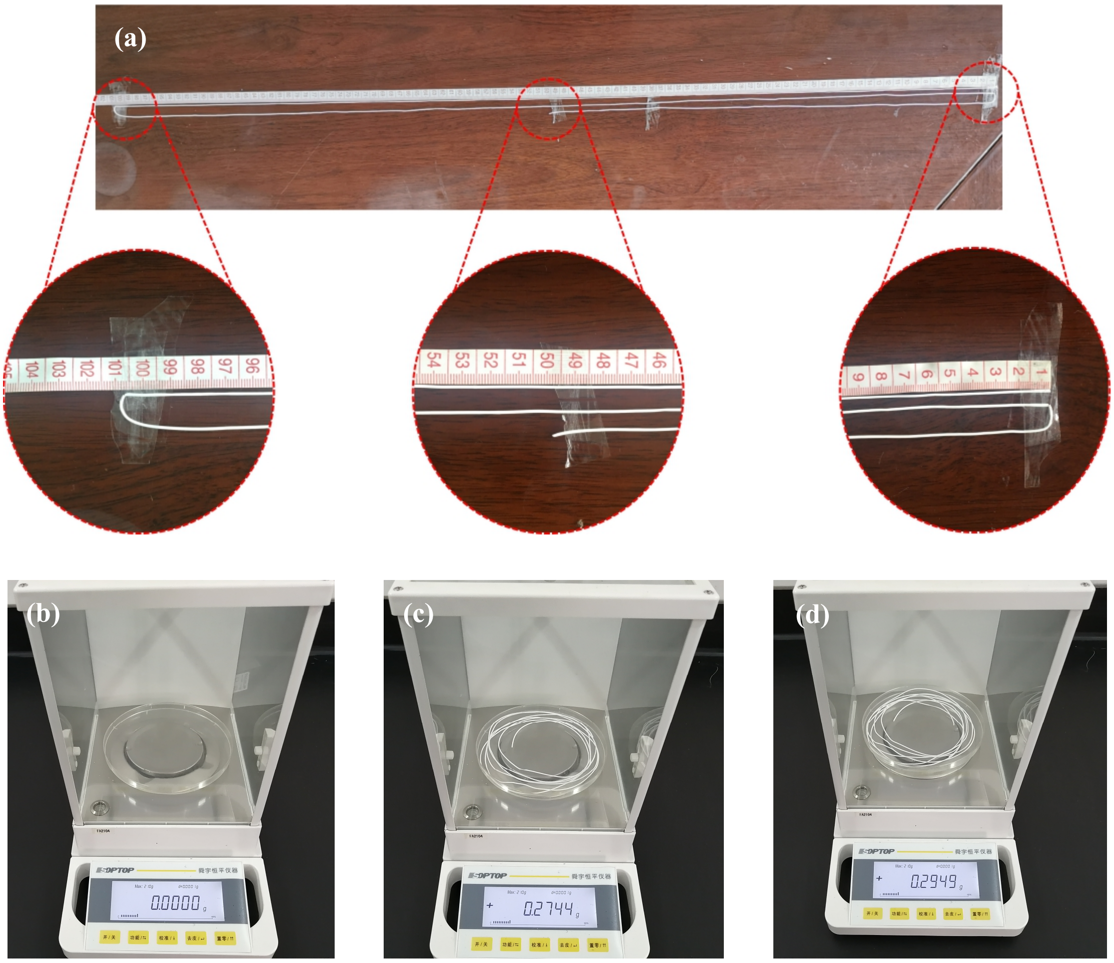

Figure S3. The length and weight CA/PAA hollow fibers and CA/PAA-wrapped SF aerogel fibers. (a) both are c.a 2.50 m length; (b-d) The weight of hollow fiber and aerogel fiber are 0.2744 g and 0.2949 g, respectively.

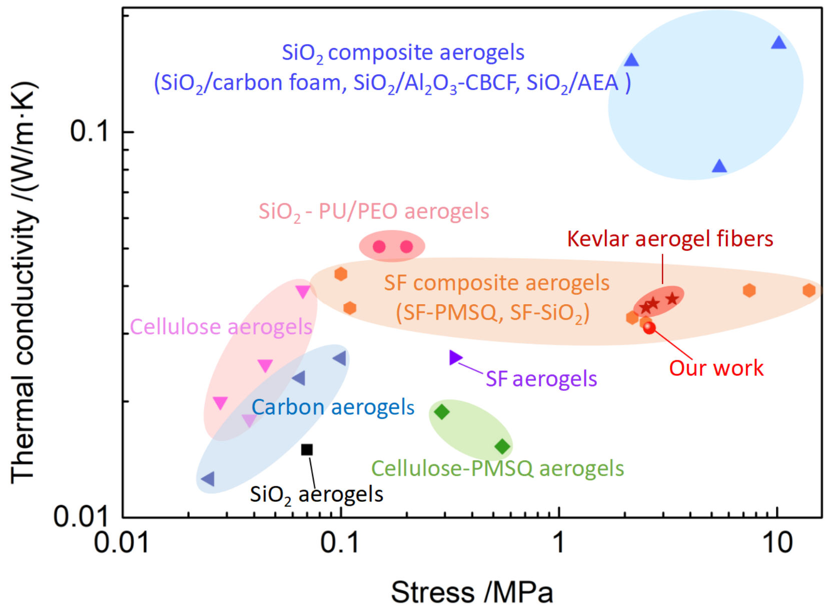

Figure S4. Thermal conductivity versus strength for the CA/PAA-wrapped SF aerogel fiber and other aerogel-based thermal insulating materials, including SiO_2 and SiO_2-based aerogels [1-6], nanocellulose and nanocellulose-based aerogels [7,8], carbon aerogels [9-11], SF and SF-based aerogels [12,13], Kevlar aerogel fiber [14].

References

1. Li, H.; Chen, Y.; Wang, P.; Xu, B.; Ma, Y.; Wen, W.; Yang, Y.; Fang, D. Porous carbon-bonded carbon fiber composites impregnated with SiO_2-Al2O_3 aerogel with enhanced thermal insulation and mechanical properties. *Ceram. Int.* **2018,** *44*, 3484-3487.

2. Demilecamps, A.; Beauger, C.; Hildenbrand, C.; Rigacci, A.; Budtova, T. Cellulose–silica aerogels. *Carbohyd. Polym.* **2015,** *122*, 293-300.

3. Li, L.; Yalcin, B.; Nguyen, B. N.; Meador, M. A. B.; Cakmak, M. Flexible nanofiber-reinforced aerogel (xerogel) synthesis, manufacture, and characterization. *Acs. Appl. Mater. Inter.* **2009,** *1*, 2491-2501.

4. Liu, H.; Li, T.; Shi, Y.; Zhao, X. Thermal insulation composite prepared from carbon foam and silica aerogel under ambient pressure. *J. Mater. Eng. Perform.* **2015,** *24*, 4054-4059.

5. Liu, Z.-h.; Ding, Y.-d.; Wang, F.; Deng, Z.-p. Thermal insulation material based on SiO_2 aerogel. *Constr. Build. Mater.* **2016,** *122*, 548-555.

6. Zhu, J.; Hu, J.; Jiang, C.; Liu, S.; Li, Y. Ultralight, hydrophobic, monolithic konjac glucomannan-silica composite aerogel with thermal insulation and mechanical properties. *Carbohyd. Polym.* **2019,** *207*, 246-255.

7. Kobayashi, Y.; Saito, T.; Isogai, A. Aerogels with 3D ordered nanofiber skeletons of liquid-crystalline nanocellulose derivatives as tough and transparent insulators. *Angew. Chem. Int. Edit.* **2014,** *53*, 10394-10397.

8. Hayase, G.; Kanamori, K.; Abe, K.; Yano, H.; Maeno, A.; Kaji, H.; Nakanishi, K. Polymethylsilsesquioxane-cellulose nanofiber biocomposite aerogels with high thermal insulation, bendability, and superhydrophobicity. *Acs. Appl. Mater. Inter.* **2014,** *6*, 9466-9471.

9. Zhang, Q.; Hao, M.; Xu, X.; Xiong, G.; Li, H.; Fisher, T. S. Flyweight 3D graphene scaffolds with microinterface barrier-derived tunable thermal insulation and flame retardancy. *Appl. Mater. Inter.* **2017,** *9* (16), 14232-14241.

10. Ye, C.; Zhang, R.; An, Z.; Wang, B. A machinable carbon aerogel composite with a low thermal conductivity and enhanced mechanical properties. *Adv. Appl. Ceram.* **2018,** *117* (8), 468-475.

11. Zhan, H.-J.; Wu, K.-J.; Hu, Y.-L.; Liu, J.-W.; Li, H.; Guo, X.; Xu, J.; Yang, Y.; Yu, Z.-L.; Gao, H.-L. Biomimetic Carbon Tube Aerogel Enables Super-Elasticity and Thermal Insulation. *Chem* **2019**, *5*, 1-12.

12. Maleki, H.; Whitmore, L.; Hüsing, N. Novel multifunctional polymethylsilsesquioxane-silk fibroin aerogel hybrids for environmental and thermal insulation applications. *J. Mater. Chem. A* **2018,** *6*, 12598-12612.

13. Maleki, H.; Montes, S.; Hayati-Roodbari, N.; Putz, F.; Huesing, N. Compressible, Thermally Insulating, and Fire-Retardant Aerogels through Self-Assembling Silk Fibroin Biopolymers Inside a Silica Structure-An Approach towards 3D Printing of Aerogels. *Acs. Appl. Mater. Inter.* **2018,** *10*, 22718-22730.

14. Liu, Z.; Lyu, J.; Fang, D.; Zhang, X. Nanofibrous Kevlar Aerogel Threads for Thermal Insulation in Harsh Environments. *ACS nano* **2019,** *13*, 5703-5711.
